# P6 acupoint stimulation for prevention of postoperative nausea and vomiting in patients undergoing craniotomy: study protocol for a randomized controlled trial

**DOI:** 10.1186/1745-6215-14-153

**Published:** 2013-05-28

**Authors:** Jian-qin Lv, Rui-zhi Feng, Ning Li

**Affiliations:** 1 Department of Integrated Traditional and Western Medicine, West China Hospital of Si-chuan University, Chengdu 610041, China

**Keywords:** Acupoint stimulation, Nausea, Vomiting, Craniotomy

## Abstract

**Background:**

The incidence of postoperative nausea and vomiting (PONV) is 50 to 79% after neurosurgery. Our study is designed to evaluate the efficacy of pericardium 6 (P6; also known as Neiguan) acupoint stimulation versus placebo, and versus routine antiemetic for the prevention of PONV after craniotomy, as well as to compare the efficacy of invasive acupuncture with non-invasive transcutaneous electrical nerve stimulation (TENS) on P6.

**Methods/design:**

This is a single-center, prospective, double-blind, five-arm, parallel-group, randomized controlled trial (RCT). All groups will be given routine intravenous ondansetron 8 mg administered before skin closure. Upon regaining consciousness from general anaesthesia, patients will receive one of five interventions: 1) P6 acupuncture bilaterally for 30 minutes, stimulated every 10 minutes to keep *de qi* sensation; 2) sham acupuncture bilaterally for 30 minutes with no stimulation; 3) P6 stimulation via active TENS electrodes bilaterally for 30 minutes, with stimulation frequency and intensity set to when *de qi* sensation is felt; 4) sham P6 stimulation via inactive TENS electrode bilaterally for 30 minutes; and 5) usual practice of pharmacological emesis prevention. The incidence of postoperative vomiting during the first 24 hours is the main outcome. Secondary outcomes include: complete response rate, severity of nausea, total rescue metoclopramide dose used and patient satisfaction with PONV management.

**Discussion:**

The results from this study could potentially confirm that P6 acupoint stimulation is an effective adjunct to standard antiemetic drug therapy for the prevention of PONV in patients undergoing craniotomy. Our study may also confirm that conventional acupuncture is more effective than TENS.

**Trial registration:**

This study is registered with the Chinese Clinical Trial Registry: ChiCTR-TRC-13003026.

## Background

As a common complication of anesthesia and surgery, postoperative nausea and vomiting (PONV) remains problematic despite use of antiemetics. PONV delays the patient’s recovery from anesthesia, extends their hospital stay and increases overall healthcare costs [1]. Although PONV is rarely fatal, the physical act of vomiting may increase cerebral intravascular pressure or intracranial pressure, which may lead to cause severe consequences in patients who have undergone craniotomy. Studies have reported an average incidence of PONV of up to 38.3% following common surgeries [2], while in patients specifically undergoing neurosurgery, the incidence is as great as 50 to 79% [3,4]. There are no significant differences in the frequency of nausea based on the craniotomy location [5]. PONV has a multi-factor etiology and the mechanism is complex. Currently, pharmacological prophylaxis is widely used in clinical practice, and the most commonly used prophylactic antiemetics include serotonin (5-HT_3_) receptor antagonists often in combination with either droperidol or dexamethasone [6,7]. Although droperidol is very effective, it can cause death secondary to arrhythmia or QT prolongation [8]. At present, no therapy is absolutely effective at preventing PONV.

Due to the limited efficacy and many side effects of drug therapy, various non-pharmacological techniques have been used in clinical practice. These therapies include acupuncture, acupressure, transcutaneous electrical nerve stimulation (TENS), electro-acupuncture, and others. Early in 2006, the American Society of PeriAnesthesia Nurses (ASPAN) recommended pericardium 6 (P6; also known as Neiguan) acupoint stimulation (Class IIb, Level A) as a complementary intervention for PONV prophylaxis [9]. According to the theory of traditional Chinese medicine (TCM), performing surgery breaks the balanced state of the human body and disturbs the movement of both *qi* and blood. When this happens, stomach *qi* will reverse its direction and go upward, causing nausea and vomiting. The main principle of treatment in this case is to regulate the function of the stomach to avoid the adverse flow of *qi*. P6 is an acupuncture point in the meridian named Jueyin Pericardium Meridian of Hand. It is both the Luo-connecting point (the point where a collateral starts to connect a definite pair of Yin and Yang meridians, which are externally-internally related) and the eight confluent point (the point connecting the eight extra meridians with the twelve primary meridians). One of P6’s main functions is to regulate the function of the stomach to avoid the adverse flow of *qi*, thus it is an effective acupoint for preventing nausea and vomiting.

Many recent studies have supported the efficacy of P6 acupoint stimulation for preventing PONV [10-12], with very few gaining a negative result [13,14]. The various ways to stimulate the P6 acupoint include laser, electrical, transcutaneous electrical and manual stimulation. Few studies have compared the efficacy of invasive methods with non-invasive methods. Thus, the optimal method of stimulation has not been determined due to lack of evidence. Although there is a great amount of research concerning P6 acupoint stimulation for preventing PONV, only two studies focus on the role of P6 acupoint stimulation in postoperative patients following craniotomy [15,16]. Therefore, we designed a single-center, prospective, double-blind, five-arm, parallel-group, randomized controlled trial (RCT) to evaluate the efficacy of P6 acupoint stimulation as a non-pharmacological prophylaxis for PONV.

The primary objective of this study is to evaluate the efficacy of P6 acupoint stimulation versus placebo, and versus routine antiemetic for the prevention of PONV in patients undergoing craniotomy under general anesthesia. The secondary study objective is to compare the efficacy of invasive acupuncture with non-invasive TENS on P6. We hypothesize that a significantly greater percentage of patients will not vomit in the subsequent 24 hours following craniotomy in the acupoint stimulation groups. Additionally, we expect that the severity of nausea will be decreased in the acustimulation groups, based on subjective patient feedback.

## Methods/design

### Design

**Figure 1:**
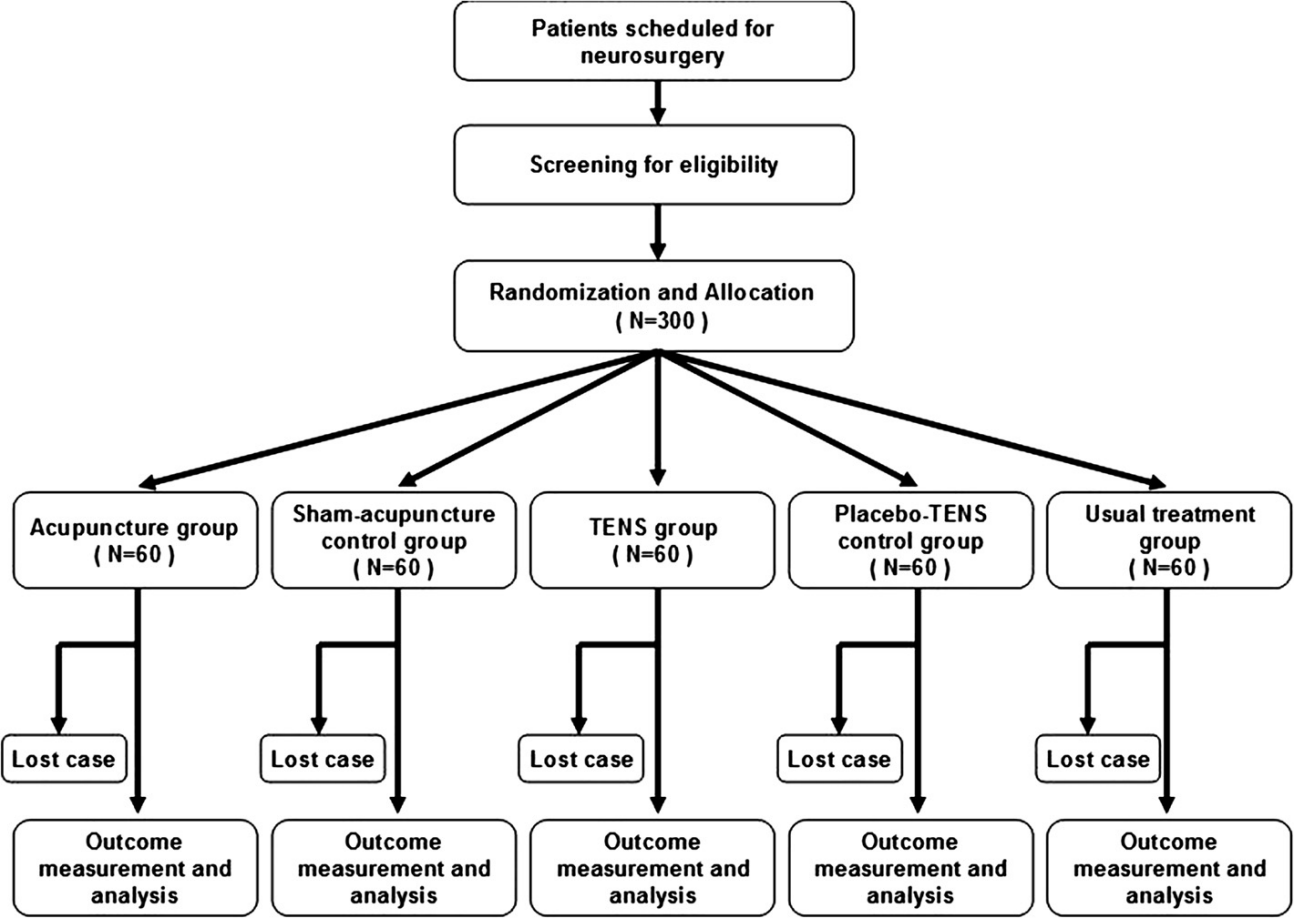
**Trial flow chart.** If a patient can not complete the study due to death, re-operation, unconsciousness or cognitive impairment, the patient will be considered a lost case and recorded as an occurrence of PONV. PONV, postoperative nausea and vomiting; TENS, transcutaneous electrical nerve stimulation.

This is a single-center, prospective, double-blind, five-arm, parallel-group, RCT. The trial protocol strictly follows the principles of the Declaration of Helsinki (version Seoul, 2008) and approval has been obtained from Si-chuan University's Ethics and Research Committee. Participants have been and will continue to be recruited from the West China Hospital of Si-chuan University (WCHSU) from January 2013 to October 2013 (Figure 1). All participants are required to give written informed consent.

### Patient population and setting

A total of 300 Chinese patients undergoing craniotomy will be sequentially recruited at the WCHSU after fulfilling the eligibility criteria and signing the informed consent. A clinical assistant with institutional review board training will be in charge of patient enrollment.

### Eligibility criteria

#### Inclusion criteria

Patients who fulfill the following conditions will be included: 1) scheduled for neurosurgery requiring opening of the cranium and dura; 2) aged between 18 and 70 years old; 3) American Society of Anesthesiologists (ASA) physical status classification of I or II; 4) undergoing general anesthesia; 5) no history of PONV or motion sickness; 6) no use of antiemetic 24 hours before surgery; 7) willing to participate; 8) no experience with acupuncture therapies; and 9) signed an informed consent form.

#### Exclusion criteria

Participants that meet any of the following criteria will be excluded: 1) nausea or vomiting 24 hours before surgery; 2) pregnant or lactating women; 3) drug or alcohol abusers; 4) recipients of chemotherapy or radiation therapy during the previous 7 days; 5) cardiac pacemaker; 6) menstruating phase of the menstrual cycle; 7) refusal to accept acupuncture and TENS treatment; 8) mental disorder; 9) history of epilepsy and still taking an antiepileptic medicine; 10) unconscious before the surgery; 11) cannot normally communicate; 12) undergoing ventricle or brainstem surgery; 13) cerebral perfusion pressure (CPP) of less than 50 mmHg or greater than 150 mmHg; 14) poorly controlled diabetes mellitus (fasting plasma glucose greater than 12 mmol/L); 15) bleeding disorders (hemophilia or afibrinogenemia); and 16) serious systemic disease (AIDS or sepsis).

### Randomization and blinding

The randomization sequence will be computer-generated by independent research staff using software called Package for Encyclopaedia Medical Statistics 3.1 (PEMS 3.1). Stratification will not be used in this trial. After randomization, group assignments will be concealed in light-proof and sealed envelopes. The included participants will be randomly enrolled by sealed envelope and assigned to the acupuncture group, sham-acupuncture control group, TENS group, placebo-TENS control group and usual treatment group, using a 1:1:1:1:1 ratio. Patient allocation will be performed by a clinical assistant, who will be trained in institutional review board policies. All study personnel including the outcome assessors, study participants, data analyst and care providers will be blinded to group assignments.

### Interventions

In this trial, we will use both invasive and non-invasive methods to stimulate the P6 acupoint as an adjunct to routine antiemetic drug therapy for the prevention of nausea and vomiting in patients undergoing craniotomy. The routine antiemetic treatment for high-risk patients at the WCHSU is a single intravenous dose of 8 mg ondansetron administered before skin closure. Therefore, neither dexamethasone nor droperidol will be used in our study.

To date, the optimal timing of acustimulation for preventing PONV has not been determined. In our study, the intervention is started right after the patient regains consciousness from anesthesia for two reasons. First, in the TCM theory, it is important to locate the right acupoint with the *de qi* sensation (the sensation of numbness, heaviness or distention associated with correct identification of an acupuncture point) to enhance efficacy, and this cannot be accomplished when the patient is unconscious. Second, a recent RCT shows that postoperative acustimulation is more effective in reducing PONV [17].

In preparation for surgery, all patients will receive a standard anesthesia and analgesia regimen specific to neurosurgical procedures. They will fast for 8 hours before general anesthesia. Standard monitors will be used to record patients' electrocardiogram, pulse oximetry, invasive arterial blood pressure and end tidal CO_2_. Induction of anesthesia will be achieved with midazolam 0.05 mg/kg, sufentanil 0.3 μg/kg, atracurium 0.15 mg/kg and propofol 2 mg/kg. When endotracheal intubation and gastrointestinal decompression with either an orogastric or nasogastric tube are undertaken, the anesthesia will be maintained with 50% nitrous oxide and 3% sevoflurane, and intermittent sufentanil 0.2 μg/kg and atracurium 0.1 mg/kg. Intravenous ondansetron 8 mg is administered as a routine antiemetic treatment for each patient before skin closure. Fentanyl 25 to 50 μg intravenous boluses are used as needed for postoperative, patient-controlled intravenous analgesia (PCIA). After surgery, patients will be continually monitored in the post-anesthesia care unit (PACU). Tracheal extubation will be undertaken when patients regain consciousness from anesthesia. Intramuscular metoclopramide 10 mg will be used as a standard rescue antiemetic and administered at the patient’s request.

**Figure 2:**
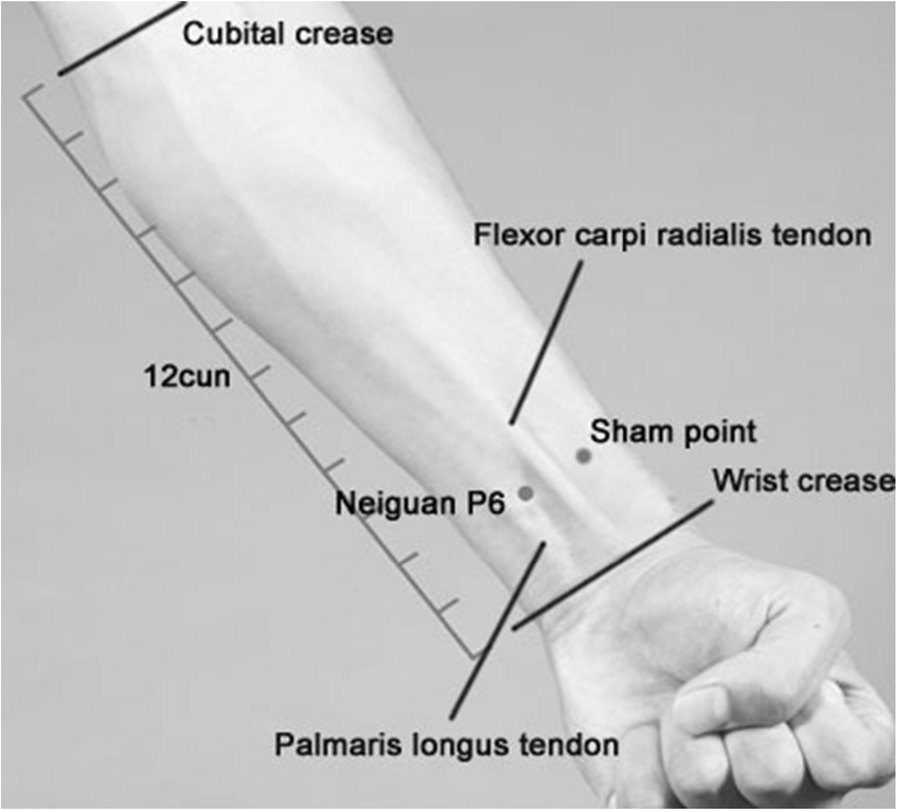
Location of P6 acupoint and sham acupoint.

When the patients regain consciousness from anesthesia and are able to communicate, the intervention will begin. All participants in our study will receive the intervention only once. In preparation for acustimulation, the patient will be placed in the best posture for P6 acupuncture, with their forearms fully exposed and with their palms facing upward. A fourth-year acupuncture resident will locate the P6 acupoint bilaterally (Figure 2) and perform the interventions specified in Table 1. P6 is an important acupuncture point in the Jueyin Pericardium Meridian of Hand. The location of P6 is 2 cun (a cun is a measurement used in acupuncture and is equivalent to the width of the distal interphalangeal joint of the first finger) above the transverse crease of the wrist, between the tendons of palmaris longus and flexor carpi radialis.

**Table 1:** Details of interventions

**Group names**	**Acupoints**	***De qi *sensation**	**Interventions**
Acupuncture group	P6 (Neiguan) bilaterally	Yes	P6 at both sides is punctured perpendicularly to a depth of 20 mm. Needles are retained for 30 minutes and stimulated every 10 minutes to keep the *de qi* sensation.
(group A)			
Sham-acupuncture control group	Sham points bilaterally	No	The sham point is punctured to a perpendicular depth of 5 mm bilaterally. Needles are retained for 30 minutes and no stimulation is performed during this duration.
(group SA)			
TENS group	P6 (Neiguan) bilaterally	Yes	P6 acupoint at both sides is stimulated by active self-adhesive electrodes for 30 minutes. The stimulation frequency is set on 2 to 100 hertz and intensity varies until the patients continue to feel the *de qi* sensation.
(group T)			
Placebo-TENS control group	P6 (Neiguan) bilaterally	No	Inactive self-adhesive electrodes are placed at the P6 acupoint bilaterally for 30 minutes.
(group PT)			
Usual treatment group	—	—	—
(group U)			

TENS, transcutaneous electrical nerve stimulation.

For the acupuncture group (group A), after skin cleaning with 75% alcohol swab, sterile and disposable stainless steel needles (0.25 × 25 mm, Wuxi Jiajian, Jiangsu, China) are quickly and perpendicularly inserted into the skin at P6 acupoints bilaterally to a depth of 20 mm. In this group, downward pressure and upward lifting combined with twirling the needle will be used to achieve *de qi* sensation. The needles will be kept in place for 30 minutes and manipulated manually every 10 minutes to maintain the *de qi* sensation. When the treatment time is over, all needles will be carefully taken out and the puncture sites will be covered with sterile swabs to avoid bleeding.

For the sham-acupuncture control group (group SA), sham points are used, which are superficial, non-acupoints on the radial side of each wrist, 15 mm away from each P6 acupoint (Figure 2). After skin cleaning with 75% alcohol swab, sterile and disposable stainless steel needles (0.25 × 25 mm, Wuxi Jiajian, Jiangsu, China) are quickly and perpendicularly inserted into the skin at sham acupoints bilaterally to a depth of 5 mm. The *de qi* sensation is not required in this group. The needles will be retained for 30 minutes as with the group A, but will not be stimulated or manipulated for the duration. After 30 minutes, the same method will be used to take out the needles as in the group A.

**Figure 3:**
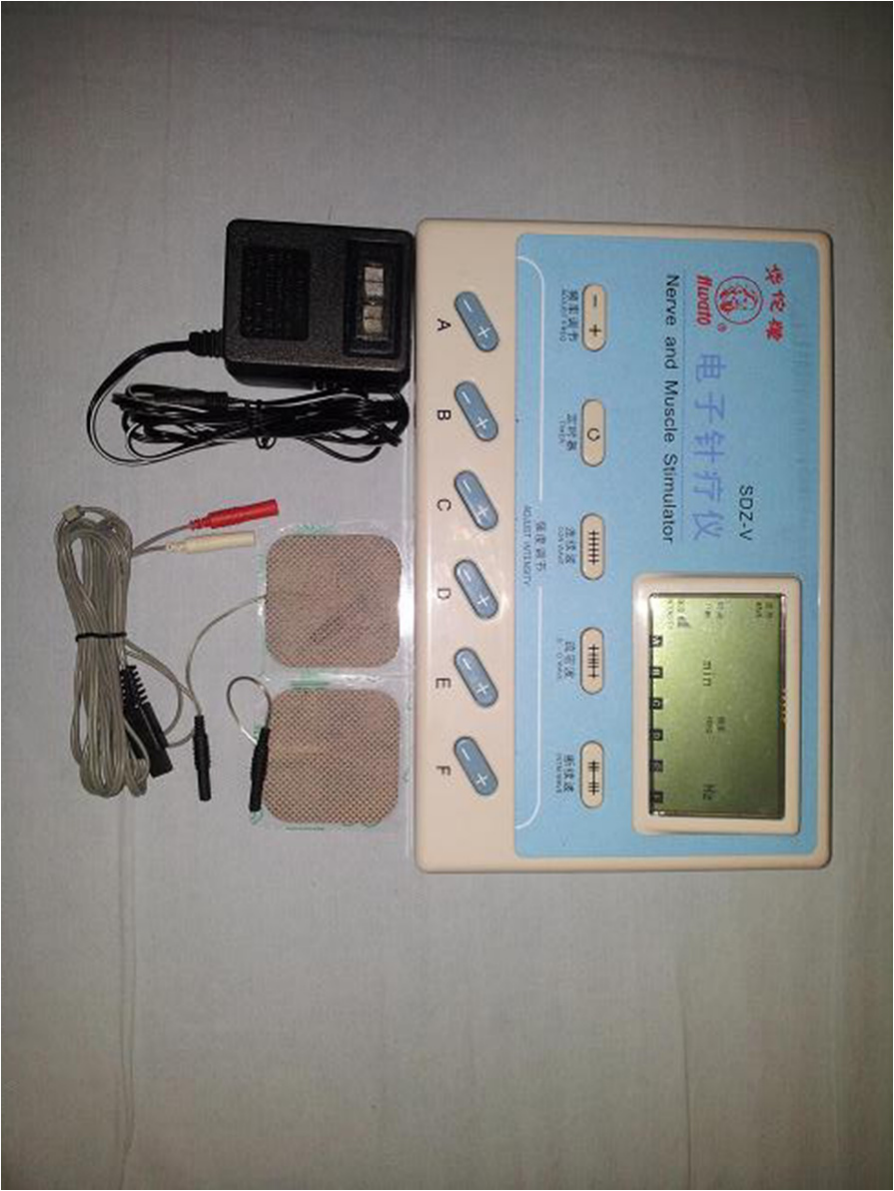
The SDZ-V nerve and muscle stimulator (Hwato, Jiangsu, China) and self-adhesive electrodes.

For the TENS group (group T), the patients' forearms are cleaned with 75% alcohol swabs and self-adhesive electrodes are placed over P6 acupoints bilaterally. SDZ-V nerve and muscle stimulators (Hwato, Jiangsu, China) are used for TENS (Figure 3). The treatment time is also 30 minutes, using dilatational wave. The stimulation frequency is set on 2 to 100 hertz and intensity varies until the patients feel the *de qi* sensation continuously. The screen on each stimulator is covered with 6 × 8 cm black tape so that no one will see the screen and know whether the stimulator is on or off. When the treatment time is complete, all self-adhesive electrodes will be removed.

For the placebo-TENS control group (group PT), patients have self-adhesive electrodes placed over P6 acupoints bilaterally, and are connected to the SDZ-V nerve and muscle stimulators for 30 minutes. However, as a placebo control group, the stimulators will not be turned on and there will be no electrical stimulation during the treatment. In order to blind patients in this group, each participant will be informed that special acupoint stimulation is used in the treatment which cannot be felt by human sensory perception. After 30 minutes, all self-adhesive electrodes will be removed as in the group T.

For the usual treatment group (group U), no interventions are undertaken. Patients in this group will be observed by the researchers as a blank control, which will provide a baseline postoperative vomiting rate and nausea score in this study using the same drug prophylaxis.

### Outcome measurement

In our study, outcomes are collected based on previously designed PONV studies [18]. The whole study period covers 24 hours after the surgery. A separate research nurse, who is not involved in the management of patients, will record anesthesia time, surgery time, endotracheal intubation time, patient demographics and preoperative data for each patient. Demographic and preoperative data will include: age, gender, race, weight, height, acupuncture experience, smoking history and CPP. Another blinded observer will record the postoperative data, which will include administration of a rescue antiemetic, patient satisfaction with PONV management, and episodes of vomiting or nausea at 2 hours, 6 hours and 24 hours postoperatively. The physicians will record the use time and dosage of rescue antiemetic for each patient when they request rescue therapy, and the records will be handed over to the observer for assessment. Nausea is a subjective sensation which should be evaluated by the patient, not by the observer. Thus, the observer will request the patients to evaluate their nausea score using a standard visual analogue scale (VAS) (0, no nausea at all; 10, worst imaginable nausea). Vomiting, which is defined as the ejection of contents of the stomach through the mouth, will be reported by the patients and assessed by this blinded observer. Patient satisfaction is not an easy concept to measure. Until recently, there has been no consensus on how to measure this concept. Since satisfaction is multi-faceted and subjective, a questionnaire cannot cover every aspect of a patient’s concerns. Therefore, in this study, patients will be asked about satisfaction with their PONV treatment at 24 hours postoperatively using another 0 (very dissatisfied) to 10 (very satisfied) scale.

The incidence of postoperative vomiting within 24 hours after craniotomy across the five groups is the main outcome we are studying. The secondary outcomes are: 1) proportion of participants with a complete response (no vomiting, no nausea, no rescue antiemetic) during 0 to 2 hours, 2 to 6 hours, 6 to 24 hours and 0 to 24 hours after craniotomy under general anesthesia; 2) assessment of nausea score (severity of nausea) during 0 to 2 hours, 2 to 6 hours and 6 to 24 hours after craniotomy under general anesthesia; 3) assessment of total rescue antiemetic dosage during 0 to 24 hours after craniotomy under general anesthesia; and 4) assessment of satisfaction score (patient satisfaction with PONV management) at 24 hours postoperatively.

### Sample size calculation and statistical analysis

A German prospective observational study published in 2011 demonstrated an overall incidence of PONV in 47% of patients after craniotomy under general anesthesia [4]. The sample size is determined by using PEMS 3.1 with α = 0.05 (two-sided) and β = 0.1 (90% power). In order to demonstrate a 30% absolute reduction in the incidence of PONV, the sample size will be 49 patients for each group. Considering the potential loss and attrition, 60 patients per group, or a total of 300 patients, should be reasonable. If a patient can not complete the study due to death, re-operation, unconsciousness or cognitive impairment, the patient will be considered a lost case and recorded as an occurrence of PONV.

All data will be analyzed by a blinded statistician using PEMS 3.1 at a separate location from the WCHSU. The intention-to-treat principal will be used in the data analysis. Baseline data will be collected and compared first. Different statistics are presented differently, for example, continuous data is presented as mean (± standard deviation). Chi-square test is used to compare the incidence of postoperative vomiting, complete response rate, sex difference and other nominal data. Conversely, the Kruskal-Wallis test is used to compare the nausea score, satisfaction score and antiemetic dosage. Additionally, the Nemenyi test and Scheffé’s method are used for multiple comparisons between groups. A *P* value <0.05 is considered statistically significant.

## Discussion

This study could potentially confirm that P6 acupoint stimulation is an effective adjunct to the standard antiemetic drug therapy for the prevention of PONV in patients undergoing craniotomy. Our study may also confirm that conventional acupuncture is more effective than TENS. The reason why we chose 2 hours, 6 hours and 24 hours postoperatively as the observation points are because: 1) volatile anesthetics may be the main cause for early (within 2 hours after the surgery) but not delayed postoperative nausea and vomiting [19]; 2) the half-life of ondansetron is approximately 6 hours based on pharmacokinetics [20]; and 3) PONV is defined as vomiting and/or nausea occurring within 24 hours after surgery [21].

Previous studies have not indicated whether the duration of P6 acupoint stimulation alters its effect on PONV. From our point of view, a long duration of acupoint stimulation will increase patients’ discomfort and pain, especially for those in the acupuncture group. However, if the stimulation time is too short, the therapeutic effect of acupuncture on PONV will be uncertain. In our study, we believe the most reasonable duration of P6 acupoint stimulation is 30 minutes, which is the common acupuncture treatment time at WCHSU.

The most difficult part in this research will be to blind the patients in different treatment groups. Recruiting patients with no experience of any kind of acupuncture therapies is part of the basic method, since patients who have not experienced *de qi* sensation are easier to blind. Furthermore, different sham groups are set up according to different stimulation methods. Acupuncture is an invasive method and we chose to blind the patients by superficially acupuncturing the sham point, which has been commonly used as an invasive control condition in several studies [22-24]. TENS is used as the non-invasive method and we chose to blind the patients by using inactive self-adhesive electrodes. In order to better blind the patients using the non-invasive methods, each participant will be informed that special acupoint stimulation is used in the treatment which cannot be felt by human sensory perception.

## Trial status

This trial is currently recruiting participants.

## Abbreviations

ASA: American Society of Anesthesiologists; ASPAN: American Society of PeriAnesthesia Nurses; CPP: Cerebral perfusion pressure; P6: Pericardium 6; PACU: Post-anesthesia care unit; PCIA: Patient-controlled intravenous analgesia; PEMS: Package for Encyclopaedia Medical Statistics; PONV: Postoperative nausea and vomiting; RCT: Randomized controlled trial; TCM: Traditional Chinese medicine; TENS: Transcutaneous electrical nerve stimulation; VAS: Visual analogue scale; WCHSU: West China Hospital of Si-chuan University.

## Competing interests

The authors declare that they have no competing interests.

## Authors’ contributions

J-QL, R-ZF and NL all contributed to the design and development of the study protocol. J-QL prepared the initial draft of the manuscript. NL was the general supervisor for this research. All authors critically reviewed the content and approved the final version.
